# Various Novel Colistin Resistance Mechanisms Interact To Facilitate Adaptation of Aeromonas hydrophila to Complex Colistin Environments

**DOI:** 10.1128/AAC.00071-21

**Published:** 2021-06-17

**Authors:** Junqi Liu, Gang Xiao, Wangping Zhou, Jun Yang, Yang Wang, Yong Wu, Xiaojun Cheng, Zhiliang Sun

**Affiliations:** a College of Veterinary Medicine, Hunan Agricultural University, Changsha, Hunan, China; b Veterinary Drug Laboratory, Hunan Institute of Animal and Veterinary Science, Changsha, Hunan, China; c Hunan Engineering Research Center of Veterinary Drug, Changsha, Hunan, China; d College of Veterinary Medicine, China Agricultural University, Beijing, China

**Keywords:** A*eromonas hydrophila*, colistin resistance, EnvZ/OmpR, gene*3832*, *mlaF*

## Abstract

Aeromonas hydrophila, a heterotrophic and Gram-negative bacterium, has attracted considerable attention owing to the increasing prevalence of reported infections. Colistin is a last-resort antibiotic that can treat life-threatening infections caused by multidrug-resistant Gram-negative bacteria. However, the mechanisms underlying colistin resistance in A. hydrophila remain unclear. The present study reveals four novel colistin resistance mechanisms in *A. hydrophila*: (i) EnvZ/OmpR upregulates the expression of the *arnBCADTEF* operon to mediate lipopolysaccharide (LPS) modification by 4-amino-4-deoxy-l-arabinose, (ii) EnvZ/OmpR regulates the expression of the autotransporter gene*3832* to decrease outer membrane permeability in response to colistin, (iii) deletion of *envZ/ompR* activates PhoP/PhoQ, which functions as a substitute two-component system to mediate the addition of phosphoethanolamine to lipid A via pmrC, and (iv) the *mlaF_D173A_* mutant confers high-level colistin resistance via upregulation of the Mla pathway. The EnvZ/OmpR two-component system-mediated resistance mechanism is the leading form of colistin resistance in *A. hydrophila*, which enables it to rapidly generate low- to medium-level colistin resistance. As colistin concentrations in the environment continue to rise, antibiotic resistance mediated by EnvZ/OmpR becomes insufficient to ensure bacterial survival. Consequently, *A. hydrophila* has developed an *mlaF* mutation that results in high-level colistin resistance. Our findings indicate that *A. hydrophila* can thrive in a complex environment through various colistin resistance mechanisms.

## INTRODUCTION

Aeromonas hydrophila-associated diseases, such as hemorrhagic septicemia in fish and diarrhea and gastroenteritis in humans (1–4), are currently attracting global attention due to the increasing prevalence of reported infections. Therefore, the development of appropriate prevention or control methods is necessary. In the absence of effective vaccines, pharmacotherapy remains the primary method to contain bacterial infections. Colistin, a cationic antimicrobial peptide that disrupts the bacterial outer membrane by binding to the lipid A portion of lipopolysaccharides (LPS), is considered a last resort for treating multidrug-resistant Gram-negative bacterial infections (5–7).

Resistance to colistin is often caused by alterations of LPS molecules that form the outer layer of the outer membrane and that serve as initial targets of colistin (8). Two-component systems (TCSs) might catalyze lipid A modifications by phosphoethanolamine (PEtN) and 4-amino-4-deoxy-l-arabinose (l-Ara4N), which results in a reduction in the net negative charge of the outer membrane that reduces the affinity of colistin for its target (9). The addition of l-Ara4N and PEtN to the lipid A component of LPS is mediated by the *arnBCADTEF* operon and *pmrC* (also known as *eptA*), respectively (10, 11). Various mechanisms of resistance to colistin have been detected in some Gram-negative bacteria. Klebsiella pneumoniae resistance to colistin is mediated by mutations in the TCSs *pmrA/pmrB*, *phoP/phoQ*, and *crrA/crrB*, which leads to further upregulation of the *arnBCADTEF* operon (12–17). Inactivation or downregulation of the *mgrB* gene confers colistin resistance (18–23). Meanwhile, three colistin resistance mechanisms have been proposed in Acinetobacter baumannii: (i) mutations within one of the lipid A biosynthesis genes, *lpxA*, *lpxC*, or *lpxD*, resulting in the loss of lipid A (8, 24), (ii) mutations in and/or overexpression of *pmrA/pmrB*, resulting in modification of the lipid A moiety of LPS (25–28), and (iii) galactosamine addition to lipid A phosphates, leading to a reduced negative surface charge (29). Colistin resistance in Pseudomonas aeruginosa and Salmonella enterica involves LPS modification via addition of l-Ara4N and PEtN (30–33).

Here, we elucidated the colistin resistance mechanisms in *A. hydrophila*, showing that EnvZ/OmpR is associated with resistance by regulating the *arnBCADTEF* operon and the autotransporter gene*3832*, while the *mlaF* mutant confers a high level of resistance. Bacteria flexibly regulate these mechanisms based on changes in the external environment to enhance survivability. A better understanding of how colistin resistance in *A. hydrophila* is generated lays a foundation for the development of prevention, control, and treatment strategies.

## RESULTS

### Serial passage of clinical colistin-susceptible *A. hydrophila* strain WCX23 on tryptic soy agar with increasing colistin creates highly colistin-resistant strain 23-C-23.

The *A. hydrophila* strain WCX23, which was responsible for a fatal diarrhea outbreak in farm-raised Deinagkistrodon acutus snakes in China, is also pathogenic to mammals (34). In the current study, the colistin-susceptible (Cst^s^) strain WCX23 (colistin MIC, 1 mg/liter) was serially passaged on Trypticase soy agar (TSA) with increasing concentrations of colistin sulfate to create a colistin-resistant (Cst^r^) strain. MIC assays showed that 23 transfers (23-C-23, MIC = 256 mg/liter) developed high-level colistin resistance (Table 1). Additionally, the colistin MIC of 23-C-23 remained stable after 30 serial passages without colistin (23-C-23-W30) (Table 1). After 23 passages without colistin, the control WCX23-W23 strain remained susceptible to colistin (Table 1). Resistance testing indicated that WCX23 and 23-C-23 were similarly susceptible to several other antibacterial agents (Table S1). Therefore, the only difference was that 23-C-23 was 256-fold more resistant to colistin than was WCX23.

**TABLE 1:** Colistin MICs of *A*. *hydrophila* strains

Strain	Description	Colistin MIC (mg/liter)
WCX23	*A. hydrophila* strain isolated from snake with diarrhea	1
23-C-13	WCX23 for 13 passages with colistin	64
23-C-23	WCX23 for 23 passages with colistin	256
23-C-23-W30	23-C-23 for 30 passages without colistin	256
WCX23-W23	WCX23 for 23 passages without colistin	1
23-C-23/mlaF	Transformation of wild-type mlaF into 23-C-23	16
WCX23/mlaF_D173A_	Transformation of mlaF_D173A_ into WCX23	64
23-C-23:ΔenvZ/ompR	EnvZ/ompR knockout from 23-C-23	8
23-C-23:CΔenvZ/ompR	EnvZ/ompR complementation from 23-C-23:ΔenvZ/ompR	256
23-C-23:Δgene*3832*	gene*3832* knockout from 23-C-23	16
23-C-23:CΔgene*3832*	gene*3832* complementation from 23-C-23:Δgene*3832*	256
23-C-23:ΔphoP/phoQ	PhoP/phoQ knockout from 23-C-23	256

### Mutation in *mlaF* contributes to high-level colistin resistance by upregulating the Mla pathway.

We determined and compared the genome sequences of the *A. hydrophila* strains WCX23 and 23-C-23 to identify genomic variations caused by colistin. The isogenic pair, WCX23 and 23-C-23, possessed essentially identical genomes, consisting of a chromosome and a plasmid. A single nucleotide polymorphism (SNP) representing one nonsynonymous mutation at 518 bp (c. A518C) was detected within *mlaF*. This mutation led to a replacement of the negatively charged aspartic acid at codon 173 (p. D173A) in WCX23 by the nonpolar amino acid alanine in 23-C-23. *mlaF*, which encodes an ABC transporter ATP-binding protein, is involved in lipid transport and is a component of the Mla pathway (phospholipid transport system) (35–37).

We then investigated whether the *mlaF* mutation contributed to colistin resistance. The wild-type *mlaF* gene cloned from WCX23 was transformed into colistin-resistant 23-C-23 (23-C-23/mlaF). Colistin resistance was 16-fold lower in 23-C-23/mlaF than in colistin-resistant 23-C-23 cells (Table 1). We also constructed loci harboring nonsynonymous *mlaF* mutations and introduced them into the colistin-susceptible strain WCX23 (WCX23/mlaF_D173A_). This resulted in a 64-fold increase in resistance to colistin on the WCX23 background (Table 1). These data suggest that *mlaF_D173A_* is responsible for colistin resistance in *A. hydrophila*.

**FIG 1:**
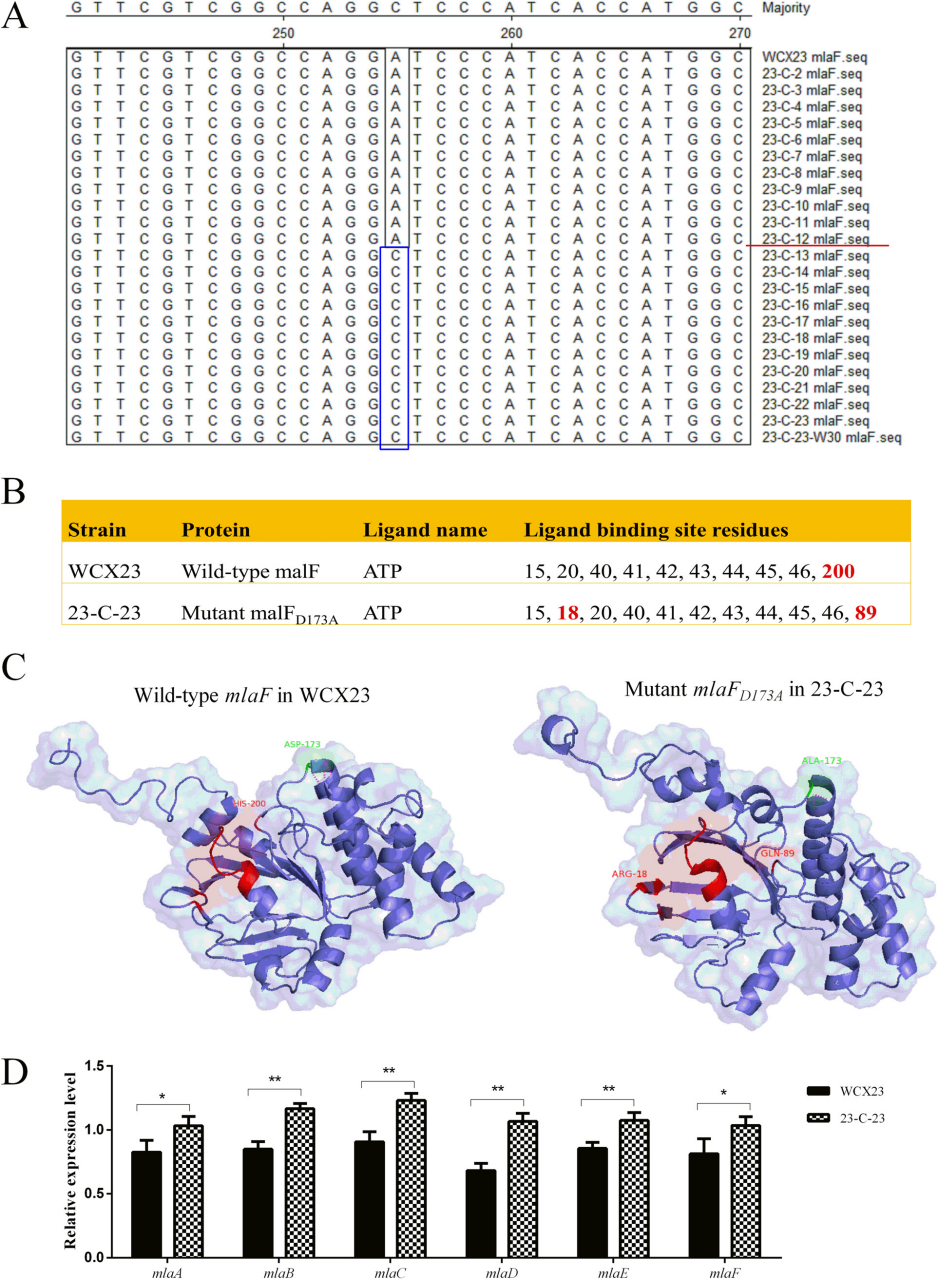
Sequencing, protein modeling, and mRNA expression of *mlaF*. (A) Nucleotide sequences were aligned with *mlaF* from WCX23 and all passaged strains in Clustal W, using MegAlign version 7.1. After 13 passages (23-C-13), nucleotide A mutated into C, and this mutation was stably maintained in 23-C-23 and 23-C-23-W30. (B) ATP-binding site residues in wild-type mlaF and mutant mlaF_D173A_. (C) i-Tasser homology modeling analysis of wild-type *mlaF* in WCX23 and *mlaF_D173A_* in 23-C-23. Aspartic acid (ASP-173) and alanine acid (ALA-173) in WCX23 and 23-C-23, respectively, are green. Polar contacts with other atoms in these two amino acids are magenta labels. ATP-binding site residues are red. (D) Expression of the Mla pathway genes in WCX23 and 23-C-23 obtained via qRT-PCR. Error bars represent the standard deviations of three biological replicates. Statistical analysis was performed using a two-tailed Student's *t* test. *, *P* value < 0.05; **, *P* value < 0.01.

To further confirm the SNP at the *mlaF* locus and the passage of the colistin-induced strain in which the *mlaF* mutation was generated, we amplified *mlaF* from all passaged strains using PCR, then sequenced the amplicons using the Sanger method. We found that the *mlaF_D173A_* mutation occurred during passage 13 (23-C-13; colistin MIC, 64 mg/liter) (Fig. 1A), which had high-level colistin resistance (Table 1). The *mlaF_D173A_* mutation was maintained in 23-C-23 after 30 serial passages without colistin (23-C-23-W30; Fig. 1A), indicating its stability in a strain with high colistin resistance even in the absence of colistin.

**FIG 2:**
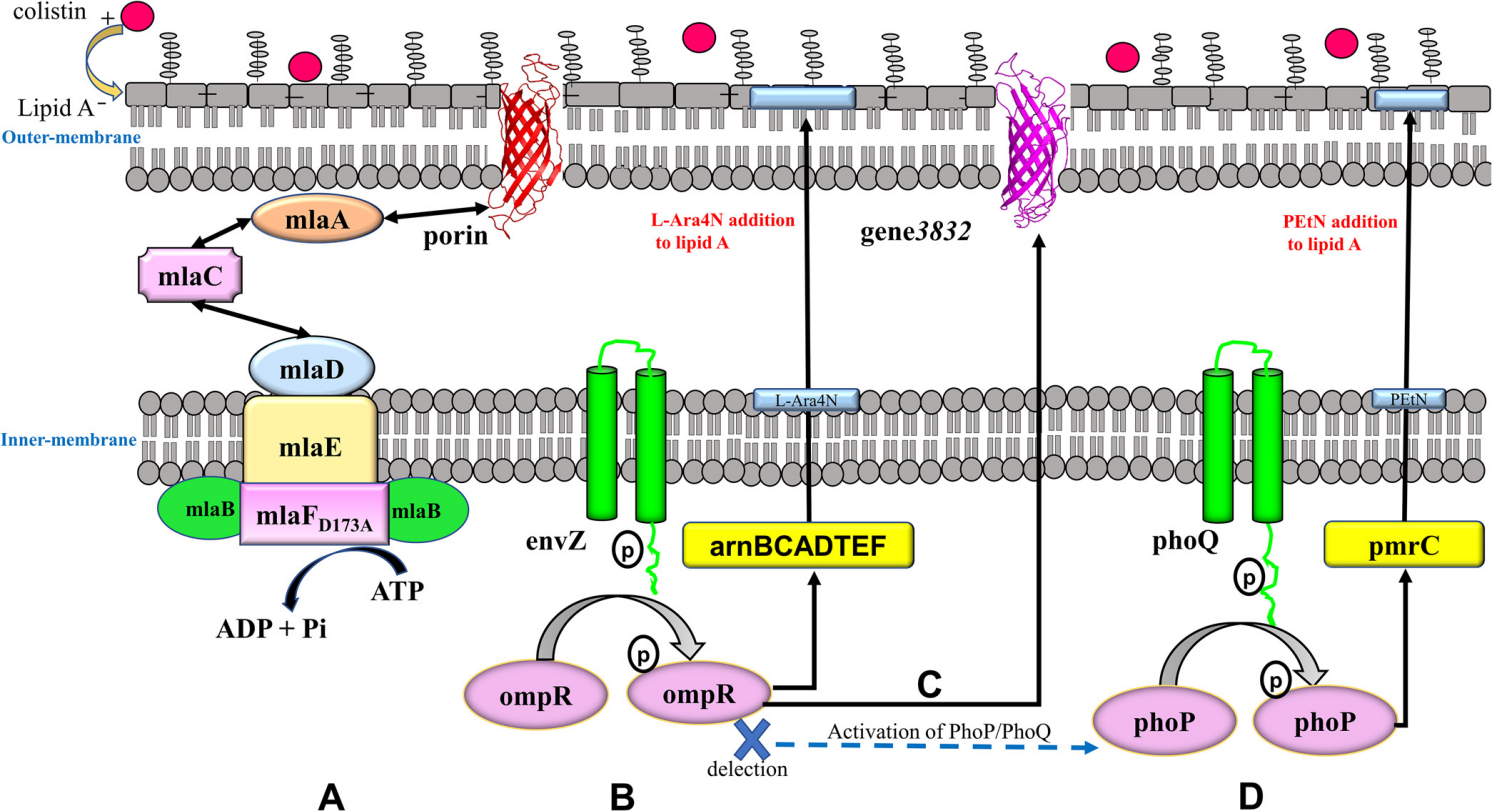
Colistin resistance mechanisms in *A. hydrophila*. (A) MlaF_D173A_ resulted in the upregulation of the Mla pathway, thus strengthening the barrier function of the outer membrane. (B) When colistin is present in the medium surrounding *A. hydrophila*, *envZ* actively undergoes auto-phosphorylation and then transfers its phosphoryl groups to the *ompR* (35, 38). The phosphorylated *ompR* activates the *arnBCADTEF* operon, which collectively modifies lipid A with l-Ara4N. (C) In response to increasing external colistin, phosphorylation of *ompR* inducted upregulation of gene*3832*, resulting in decreased permeability of the outer membrane. (D) phoP/phoQ was activated owing to the deletion of *EnvZ/OmpR*. Lipid A was modified with PEtN by *pmrC*.

We constructed structural models for wild-type *mlaF* and *mlaF_D173A_* using the i-Tasser server. The models showed that His-200 serves as the exclusive ATP-binding site residue in WCX23, whereas Arg-18 and Gln-89 were the exclusive ATP-binding site residues in 23-C-23 (Fig. 1B). Moreover, aspartic acid and alanine acid were located at position 173 of MlaF in WCX23 and 23-C-23, respectively, and the number of polar contacts with other atoms in these two amino acids was altered from four to two (Fig. 1C), suggesting that the nonsynonymous mutation had altered MlaF functionality. Moreover, reverse transcription-quantitative PCR (qRT-PCR) confirmed an increase in the expression of MlaABCDEF in the colistin-resistant mutant 23-C-23 (Fig. 1D), implying that *mlaF_D173A_* activated the Mla pathway, which maintains outer membrane lipid asymmetry via phospholipid transport (36, 38), ultimately strengthening the barrier function of the outer membrane (Fig. 2A).

### EnvZ/OmpR TCS upregulates expression of the *arnBCADTEF* operon to mediate lipid A modification by l-Ara4N.

**FIG 3:**
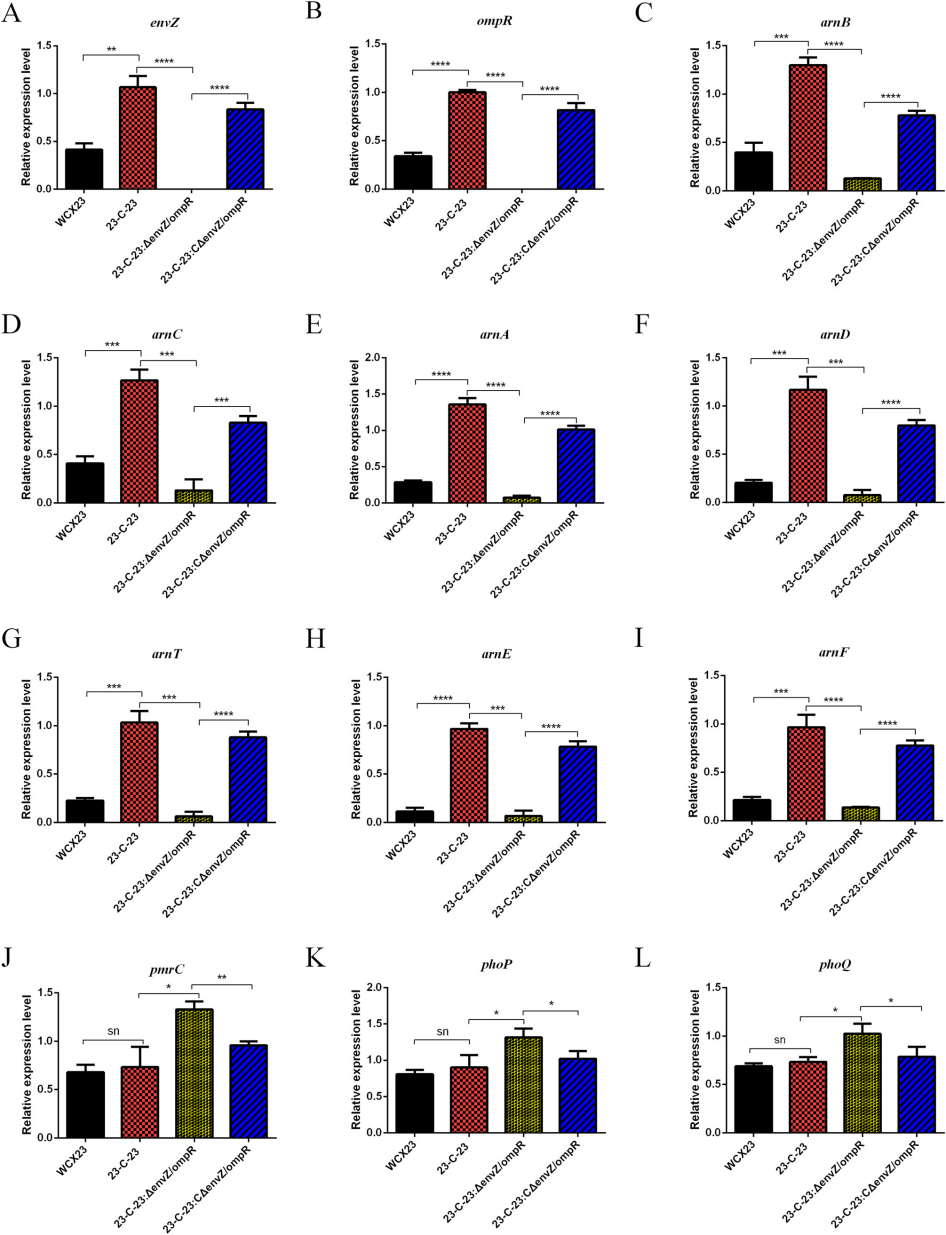
Gene expression of WCX23, 23-C-23, 23-C-23:ΔenvZ/ompR, and 23-C-23:CΔenvZ/ompR by qRT-PCR. Relative expression levels of genes were determined using the 2^−ΔΔCT^ method. Error bars represent the standard deviations of three biological replicates. Statistical analysis was performed using a two-tailed Student’s *t* test. Relative expression levels of *envZ* (A), *ompR* (B), *arnB* (C), *arnC* (D), *arnA* (E), *arnD* (F), *arnT* (G), *arnE* (H), *arnF* (I), *pm*rC (J), *phoP* (K), and *phoQ* (L) mRNA in strains. *, *P* value < 0.05; **, *P* value < 0.01; ***, *P* value < 0.001; ****, *P* value < 0.0001; ns, not significant.

TCS activation is a colistin resistance mechanism that is common among bacteria (39–42). Transcriptome analysis showed that EnvZ/OmpR was the only TCS in 23-C-23 that was significantly upregulated relative to that in Cst^s^ WCX23, and quantitative RT-PCR further confirmed that the expression of *envZ/ompR* mRNA is increased during the development of colistin resistance (Fig. 3A and B). These data imply that EnvZ/OmpR is involved in colistin resistance in *A. hydrophila*. We then knocked out *envZ/ompR* on a 23-C-23 background. The colistin MIC for 23-C-23:ΔenvZ/ompR was 32-fold lower than that for 23-C-23 (Table 1). Thus, the deletion of *envZ/ompR* in 23-C-23 significantly increased susceptibility, whereas complementation of 23-C-23:ΔenvZ/ompR with *envZ/ompR* restored resistance to colistin (Table 1). Moreover, knockout and complementation with *envZ/ompR* did not significantly alter the susceptibility to other antibiotics (Table S1). This finding underscores the importance of the EnvZ/OmpR TCS to colistin resistance in *A. hydrophila*.

The *arnBCADTEF* operon, which is involved in lipid A modification by l-Ara4N, was expressed at higher levels in 23-C-23 than in WCX23 (Fig. 3C to I), whereas that of *pmrC*, encoding lipid A PEtN transferase, did not significantly differ (Fig. 3J). These findings suggest that the *arnBCADTEF* operon-mediated addition of l-Ara4N to lipid A was the more critical LPS modification in *A. hydrophila*. To further identify whether the EnvZ/OmpR TCS contributes to colistin resistance in *A. hydrophila* by regulating *arnBCADTEF*, we compared transcriptomic differences between 23-C-23 and 23-C-23:ΔenvZ/ompR. Expression of the *arnBCADTEF* operon was obviously decreased by 5.4- to 28.8-fold in 23-C-23:ΔenvZ/ompR compared with that in the parent 23-C-23 strain, which was confirmed by qRT-PCR (Fig. 3C to I).

We characterized the lipid A modifications in these strains using matrix-assisted laser desorption ionization-time of flight (MALDI-TOF) mass spectrometry. The WCX23 showed major peaks at mass/charge ratios (*m/z*) of 1,554 and 1,570, representing a basic lipid A structure and lipid A with an additional hydroxylation, respectively (Fig. S1). Lipid A from 23-C-23 contained two additional peaks at *m/z* 1,685 and 1,701, which corresponded to an l-Ara4N residue (Δ*m/z* = +131) added to the lipid A structures at *m/z* 1,554 and 1,570 (Fig. S1). Deletion of *envZ/ompR* resulted in the loss of l-Ara4N (131 mass units; Fig. S1), while complementation of *envZ/ompR* resulted in the addition of l-Ara4N to lipid A (Fig. S1). These results further confirmed that *envZ/ompR* was responsible for colistin resistance via the *arnBCADTEF* operon, which mediates the addition of l-Ara4N to lipid A (Fig. 2B).

### EnvZ/OmpR regulates expression of autotransporter gene*3832* to decrease outer membrane permeability in response to colistin.

**FIG 4:**
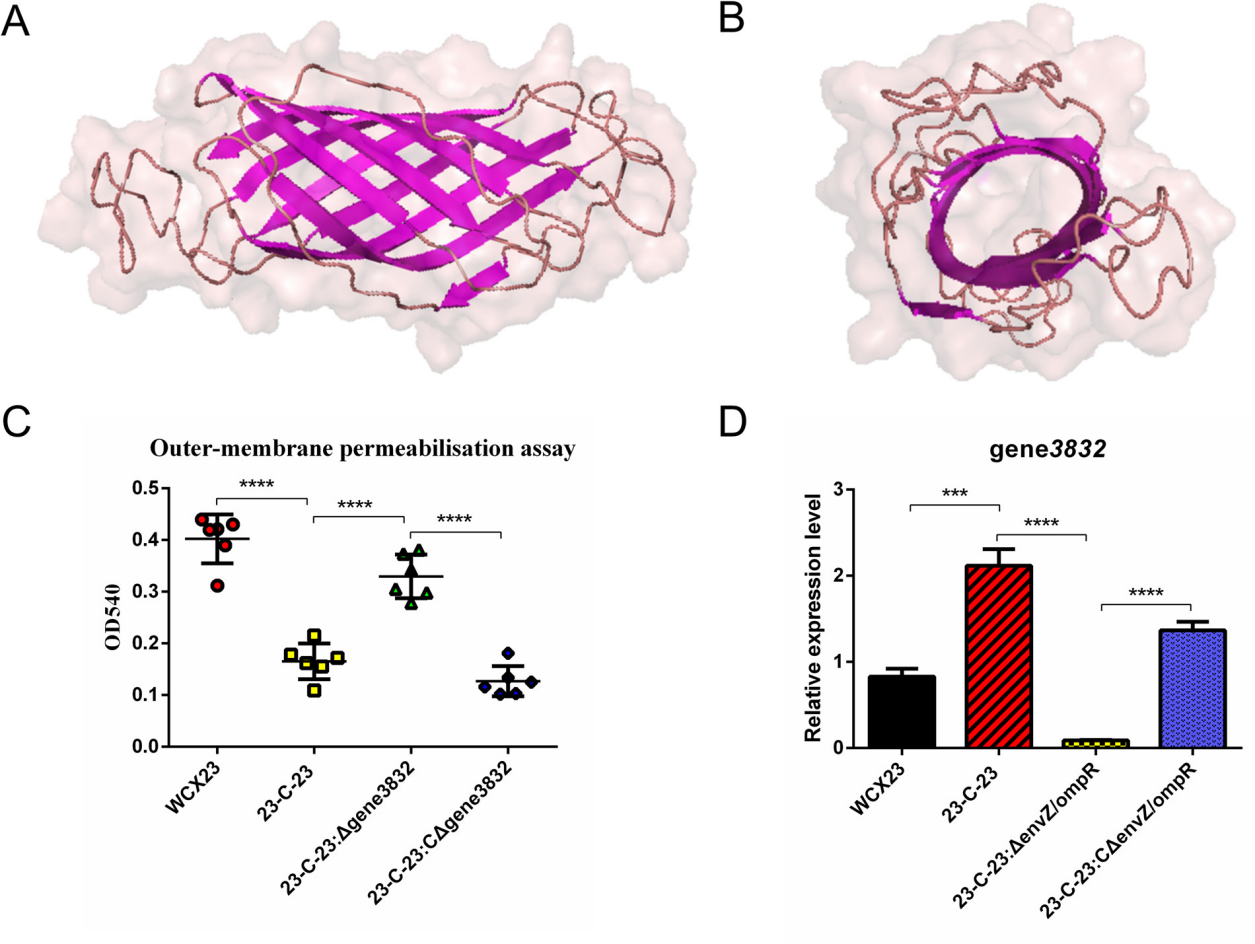
Protein modeling, outer membrane permeabilization, and mRNA expression of gene*3832*. (A) Side view of the *A. hydrophila* gene*3832*. The sheet is magenta-colored, and the loop is wheat-colored (B) Extracellular view of gene*3832*. (C) Outer membrane permeabilization between WCX23, 23-C-23, 23-C-23:Δgene*3832*, and 23-C-23:CΔgene*3832*. (D) gene*3832* expression in WCX23, 23-C-23, 23-C-23:ΔenvZ/ompR, and 23-C-23:CΔenvZ/ompR. ***, *P* value < 0.001; ****, *P* value < 0.0001.

Genome annotation predicted that gene*3832* was an autotransporter. Structural models of gene*3832* constructed using the i-Tasser server showed that gene*3832* possessed a porin-like structure and comprised β-strands arranged in β-barrels embedded in the outer membrane (Fig. 4A and B). The increase in gene*3832* expression was the third highest in the Cst^r^ strain, 23-C-23, compared with that in the Cst^s^ strain, WCX23 (Fig. S2), suggesting that gene*3832* is associated with colistin resistance in *A. hydrophila*. To test this hypothesis, the gene*3832* locus was deleted from the 23-C-23 background. The colistin MIC for 23-C-23:Δgene*3832* was decreased 16-fold compared with that for the parent 23-C-23 strain (Table 1). Complementation of gene*3832* in 23-C-23:Δgene*3832* restored high colistin resistance (Table 1). These findings confirmed that gene*3832* is involved in the colistin resistance of *A. hydrophila*.

The outer membrane is the first line of bacterial defense against antibiotics. Outer membrane permeability was reduced in 23-C-23 compared with that in WCX23 (Fig. 4C), implying that decreased molecular exchange in the outer membrane is an additional strategy for *A. hydrophila* to develop colistin resistance. Deleting gene*3832* caused an increase in the outer membrane permeability, whereas complementation with gene*3832* reduced the outer membrane permeability of 23-C-23 (Fig. 4C), suggesting that gene*3832* participates in the downregulation of outer membrane permeability.

Interestingly, gene*3832* was the most downregulated gene (236-fold decreased expression) in 23-C-23:ΔenvZ/ompR compared with that in the parent 23-C-23 strain (Fig. S2). Quantitative RT-PCR further confirmed that the deletion of *envZ/ompR* decreased the expression of gene*3832* mRNA (Fig. 4D). These findings suggest that EnvZ/OmpR is a regulator of gene*3832* transcription. Overall, EnvZ/OmpR upregulated the expression of porin gene*3832* to decrease outer membrane permeability in response to external colistin, thus reducing the colistin entry into cells, representing another colistin-resistant mechanism mediated by EnvZ/OmpR (Fig. 2C).

### Deletion of EnvZ/OmpR activates PhoP/PhoQ as a substitute TCS that mediates PEtN addition to lipid A via pmrC.

The expression of *phoP/phoQ* in *A. hydrophila* during the development of colistin resistance did not significantly change (Fig. 3K to L), indicating that PhoP/PhoQ is not involved in colistin resistance in *A. hydrophila*. Moreover, deletion of *phoP/phoQ* in 23-C-23 showed that susceptibility of the 23-C-23:ΔphoP/phoQ strain to colistin remained unchanged compared with that of 23-C-23 (Table 1). Therefore, the EnvZ/OmpR TCS functions in colistin resistance, whereas the PhoP/PhoQ TCS does not.

However, expression levels of *phoP/phoQ* and *pmrC* were higher in 23-C-23:ΔenvZ/ompR than in 23-C-23 (Fig. 3J to L), suggesting that *envZ/ompR* deletion might activate PhoP/PhoQ as a substitute TCS. PhoP/PhoQ upregulates the expression of *pmrC*, which mediates PEtN addition to lipid A, consequently leading to colistin resistance (Fig. 2D).

## DISCUSSION

The *A. hydrophila* strains in our studies were resistant to certain antibiotics, including amoxicillin, lincomycin, streptomycin, kanamycin, sulfamethoxazole, and cefradine (Table S1). We identified putative antibiotic resistance genes carried by strains via a BLAST search against the CARD (Comprehensive Antibiotic Resistance Database, http://arpcard.Mcmaster.ca). The strains were found to harbor multiple resistance genes, including beta-lactamase resistance genes (e.g., *cphA7*, *aqu1*, and *oca12* in the chromosome and *oxa17* and *cmy2* in the plasmid), aminoglycoside resistance genes (e.g., *aph(3′)-Ia*, *aph(*6*)-Id*, and *aph(3′')-Ib* in plasmid), a sulfonamide resistance gene (e.g., *sul2* in the plasmid), and macrolide resistance genes (e.g., *mrx* and *mphA* in the plasmid; Table S2). We speculated that the presence of these resistance genes provided WCX23 and 23-C-23 with resistance to specific antibiotics.

The Mla pathway has been implicated in the maintenance of outer membrane integrity (43, 44), which is a critical barrier that restricts the traffic of antibiotics into cells. Moreover, the export of *de novo* synthesized phospholipids to the outer membrane during cell growth might be driven by Mla (35). The Mla system consists of an MlaFEDB complex in the inner membrane, MlaA-porin in the outer membrane, and the shuttle protein MlaC in the periplasmic region (35–37). MlaF interacts with MlaB to comprise the cytoplasmic portion of the MlaFEDB complex (44), which drives phospholipid trafficking across the bacterial envelope to maintain outer membrane integrity. Meanwhile, MlaC ferries lipids between the outer and inner membrane complexes, and the MlaA-porin complex extracts lipids for import from the outer membrane. Herein, we confirmed that the *mlaF_D173A_* mutation was responsible for colistin resistance. We speculated that the *mlaF_D173A_* mutant might lead to enhanced ATP binding, that is, increased *mlaF* activity would serve to activate the Mla pathway, which would subsequently decrease colistin import.

We further hypothesized that various mutations might randomly occur in functional genes during the development of colistin resistance and that these mutations may either be silent or be beneficial facilitating the survival of bacteria under antibiotic pressure. Ultimately, the advantageous mutants become selected and stably inherited with increasing high-level antibiotics. We observed that only *mlaF* mutation occurred in the high-level colistin-resistant strain, suggesting that *mlaF* mutation was the consequence of evolution in response to high-level colistin.

TCSs are key players in bacterial adaptation to environmental changes. Expression of the outer membrane porins, OmpF and OmpC, is regulated by EnvZ/OmpR in some bacteria during the response to adverse environmental conditions, including changes in osmolarity and acidic pH, as well as starvation and virulence (45–49). Here, we confirmed that EnvZ/OmpR is also associated with colistin resistance in *A. hydrophila*. Moreover, in view of the absence of *ompF* or *ompC* in *A. hydrophila*, we confirmed that EnvZ/OmpR regulates the expression of autotransporter gene*3832* in response to colistin.

Antibiotics in clinical practice are commonly selected to treat bacterial infections according to agar dilution test results. Using this approach, *A. hydrophila* is classified as colistin susceptive. However, if treated with a low dose, *A. hydrophila* might develop relative colistin resistance via the EnvZ/OmpR TCS, eventually leading to therapeutic failure and the generation of a colistin-resistant strain. It is, therefore, not recommended that *Aeromonas*-associated infections be treated with colistin, which agrees with the results of previous studies (50–52).

Previous research reported that the PhoP/PhoQ TCS is associated with colistin resistance in E. coli, P. aeruginosa, Salmonella enterica serovar Typhimurium, and K. pneumoniae (14, 22, 23, 32, 39, 40). Although PhoP/PhoQ is not generally involved in colistin resistance in *A. hydrophila*, following deletion of *envZ/ompR* in *A. hydrophila*, PhoP/PhoQ assumed the task of regulating lipid A modification, thereby leading to colistin resistance. Thus, PhoP/PhoQ may function as a supplementary mechanism of colistin resistance. Therefore, bacteria flexibly adjusted various resistance mechanisms in response to a changing environment.

In terms of lipid A modification, EnvZ/OmpR and PhoP/PhoQ appear to mediate entirely different pathways. We showed that a higher level of colistin resistance was conferred on *A. hydrophila* by changes in l-Ara4N than by changes in PEtN, according to the colistin MIC of 23-C-23 and 23-C-23:ΔenvZ/ompR (with l-Ara4N- and PEtN-modified lipid A, respectively). Therefore, colistin resistance is more effective when regulated by EnvZ/OmpR than when regulated by PhoP/PhoQ in *A. hydrophila*.

## MATERIALS AND METHODS

### Strains, plasmids, and growth conditions.

The *A. hydrophila* strain WCX23 was isolated from a snake with fatal diarrhea in the city of Wangcheng, Hunan province in 2017 and was identified as ST516 via multilocus sequence typing (MLST) (34). All *A. hydrophila* isolates were maintained on TSA (Oxoid, Basingstoke, UK) or cultured in tryptone soy broth (TSB; Oxoid), supplemented with antimicrobial agents as appropriate, at 28°C. Escherichia coli strains were routinely grown in lysogeny broth (LB) or on LB agar (Oxoid), supplemented with the appropriate antibiotics as required, at 37°C. Plasmids pKD3 and pCVD442 were obtained from BioVector NTCC Inc. (Beijing, China), and pUC57-Apr, pDS132, and pUC57-sacB-Apr were donated by Y. Zhang (YiHong Biotech Ltd., Shanghai, China).

### Selection of colistin resistance.

We selected high-level colistin resistance in the susceptible (colistin MIC, 1 mg/liter) *A. hydrophila* WCX23 strain by serial daily passages on TSA with increasing concentrations of colistin sulfate. Initially, WCX23 was grown at a sub-MIC of colistin (0.5 mg/liter) at 28°C for 24 h; colonies were then transferred and streaked on the same media. Bacterial growth (including the number, morphology, and size of colonies) was observed to judge the adaptation of bacteria under drug pressure. Colistin concentrations were doubled when vigorous growth was observed. High-level resistance (colistin MIC, 256 mg/liter) that developed after 23 passages (23-C-23) was stable after 30 serial passages in TSA without antibiotics. We also passaged control WCX23 23 times without colistin. All antibiotic-induced strains were maintained separately in TSB with 30% glycerol at −80°C.

### Antimicrobial susceptibility testing.

The MIC of colistin and other antibiotics was determined using broth microdilution at 35 ± 1°C and confirmed in three independent experiments. Antimicrobial susceptibility was defined according to CLSI instructions (53) and EUCAST clinical breakpoints (http://eucast.org).

### Whole-genome sequencing and comparative genome analysis.

Genomic DNA was extracted using a bacterial DNA kit (Omega Bio-Tek Inc., Norcross, GA, USA) as per the manufacturer’s instructions. Strains were sequenced and assembled (Shanghai Majorbio Bio-pharm Technology Co., Ltd., Shanghai, China) as follows. Genomic DNA (5 μg) was sheared using g-TUBE (Covaris Inc. Woburn, MA, USA), and then a 10-kb sequencing library was constructed using standard PacBio RS sample preparation instructions and sequenced on the Pacific Biosciences RS II platform (Pacific Biosciences Inc., Menlo Park, CA, USA). A 400-bp paired-end library was also prepared from the same genomic DNA according to Illumina TruSeq DNA sample preparation recommendations and sequenced on HiSeq 2500 platforms (Illumina Inc., San Diego, CA, USA) with a read length of 150 bp. PacBio data assembled using the Hierarchical Genome Assembly Process software generated a one-contig genome and one plasmid. The genome and plasmid were further proofread using HiSeq data and Canu and SPAdes software (54). Finally, a whole-genome assembly was obtained without redundancy. Assembled scaffolds were processed using Glimmer 3.02 and GeneMarkS software to predict genes (55). Thereafter, tRNA and rRNA genes were predicted using rRNAscan-SE v2.0 and Barrnap software, respectively. Predicted proteins were compared with a nonredundant GenBank database using BLASTP, then functionally annotated using the NR, Swiss-Prot, Pfam, EggNOG, Gene Ontology, and KEGG databases, as well as manual supplementation. Clean reads of 23-C-23 were aligned with the WCX23 strain genome using Burrows-Wheeler Aligner software (56). Reads near putative insertions or deletions (InDels) were realigned using Genome Analysis Toolkit (GATK) software to eliminate false-positive SNPs. Small InDels and SNPs detected by VarScan were further filtered to generate high-confidence data. Structural variation was detected using BreakDancer software.

### Transcriptome analysis.

The concentration and purity of total RNA extracted using Qiagen RNeasy Minikits (Qiagen, Hilden, Germany) were determined using a NanoDrop 2000 spectrophotometer (Thermo Fisher Scientific Inc., Waltham, MA, USA). rRNA was removed, and cDNA libraries were prepared as previously described (57). The cDNA libraries were sequenced and analyzed (Shanghai Majorbio Bio-pharm Technology Co., Ltd.) and are described in brief below. Strand-specific cDNA libraries were generated according to the Illumina standard protocol for high-throughput sequencing and sequenced using an Illumina HiSeq. Differentially expressed genes were defined as those with a log_2_ fold change (logFC) of >1 (increased expression) or a logFC of <−1 (decreased expression), and statistical significance was set at a *P* value of <0.05.

### Determination of mRNA expression levels using qRT-PCR.

Total RNA was extracted from mid-log-phase (optical density at 600 nm [OD_600_] 0.5 ± 0.05) bacterial cultures using the Qiagen RNeasy minikit (Qiagen), according to the manufacturer’s instructions. Complementary DNA (cDNA) was generated from total RNA using a random primer hexamer. Table S3 shows the sequences of transcript-specific primers used for qRT-PCR. All reactions were performed in triplicate, and experimental data were normalized to 16S rRNA (GenBank accession number: MN901224) levels and analyzed using the 2^−ΔΔCT^ method.

### Amplification and sequencing of *mlaF* by PCR.

Fragments of the *mlaF* gene from WCX23, and all passaged strains, were amplified by PCR using the respective 5′- AGAGCGGCGCCCTGTTTAC-3′ and 5′- CCTTGCGGTTGGCGATGAT-3′ primers for mlaF-F and mlaF-R. Product sequences were determined via Sanger sequencing (BIOSUNE Biotechnology Co., Ltd., Changsha, China).

### Protein modeling.

Homology models of wild-type *mlaF*, *mlaF_D173A_*, and gene*3832* were constructed using the i-Tasser server (https://zhanglab.ccmb.med.umich.edu/I-TASSER/), and structure-based protein function annotations were predicted using COFACTOR and COACH (58–60).

### Allelic replacement of the *mlaF* mutant.

DNA fragments upstream and downstream of the mutation were amplified from 23-C-23 (WCX23) genomic DNA. *sacB*-Apr was amplified using pUC57-sacB-Apr as a template. Table S4 lists all primer pairs. The junction of the upstream fragment *sacB*-Apr and the downstream fragment was amplified via fusion PCR and cloned into pCVD442. The resulting plasmid was transformed into E. coli β2155 by electroporation and introduced into the recipient strain 23-C-23 (or WCX23) by conjugation. Transconjugants were selected on LB agar plates supplemented with apramycin. The recipient strain (23-C-23/mlaF_D173A_:sacB-Apr or WCX23/mlaF:sacB-Apr) was confirmed using PCR and sequencing.

The upstream fragment-wild-type *mlaF*-downstream fragment was amplified via PCR using WCX23 genomic DNA as a template, cloned into the suicide plasmid pDS132 harboring the chloramphenicol-resistance gene, and transformed into E. coli β2155 via electroporation to obtain the donor strain β2155/pDS132-WCX23mlaF. We similarly constructed β2155/pDS132-23-C-23mlaF_D173A_ from 23-C-23.

Conjugation was assayed in the recipient 23-C-23/mlaF_D173A_:sacB-Apr (or WCX23/mlaF:sacB-Apr) and donor β2155/pDS132-WCX23mlaF (or β2155/pDS132-23-C-23mlaF_D173A_) strains. The transconjugants were selected on LB agar plates supplemented with chloramphenicol and identified via PCR and sequencing. Colonies were selected on LB agar plates containing 10% sucrose (without NaCl) to obtain the mutant strain 23-C-23/mlaF (or WCX23/mlaF_D173A_).

### Genetic manipulations for gene knockout and complementation.

Upstream and downstream DNA fragments were amplified via PCR using Aeromonas hydrophila 23-C-23 genomic DNA as a template. The chloramphenicol-resistance gene *cmr* was amplified by PCR using pKD3 as a template. Table S5 lists all primer pairs used for gene knockout and complementation. The junction of the upstream fragment, *cmr*, and the downstream fragment was amplified via fusion PCR and cloned into pCVD442, a suicide plasmid containing the ampicillin resistance gene, to obtain recombinant plasmids that were transformed into E. coli β2155 via electroporation to obtain donor plasmids that were introduced into the recipient 23-C-23 strain via conjugation. Transconjugants were selected on LB agar plates supplemented with ampicillin (100 mg/liter) and chloramphenicol (22 mg/liter). Aeromonas hydrophila 23-C-23:ΔenvZ/ompR, 23-C-23:Δgene*3832*, and 23-C-23:ΔphoP/phoQ were confirmed by PCR and sequencing.

Upstream and downstream DNA fragments from 23-C-23 were amplified using PCR to construct the complementation strain. The apramycin resistance gene *apr* from the pUC57-Apr plasmid was amplified via PCR. The resulting products (upstream fragment-complementary gene-*apr*-downstream fragment) were connected using fusion PCR and cloned into the suicide plasmid, pCVD442. The resulting plasmid was transformed into E. coli β2155 by electroporation to obtain donor plasmids that were introduced into recipient 23-C-23:ΔenvZ/ompR or 23-C-23:Δgene*3832* strains by conjugation. Transconjugants were selected on LB agar plates supplemented with ampicillin (100 mg/liter) and apramycin (33 mg/liter). Positive clones were then selected on LB agar plates with apramycin (33 mg/liter) and chloramphenicol (34 mg/liter), respectively. The complemented strains 23-C-23:CΔenvZ/ompR or 23-C-23:CΔgene*3832* were screened by growth in LB agar plates with apramycin without chloramphenicol and were further confirmed via PCR and sequencing.

### Mass spectrometry analysis of lipid A structure.

Lipid A was isolated from strains as previously described (61) and was assessed using MALDI-TOF MS (Bruker Daltonics Inc., Billerica, MA, USA) in the negative ion mode. Data were acquired and analyzed using flexControl version 3.4 and flexAnalysis version 3.4, respectively (Bruker Daltonics).

### Outer membrane permeabilization assay.

Late-log-phase cells (1 ml) were centrifuged at 12,000 × *g* and 4°C, and the supernatant was discarded. Sedimented cells were suspended in 5 mmol/liter HEPES (pH 7.2) containing 0.1% SDS and incubated at 28°C for 10 min. Absorbance was measured at 540 nm.

### Statistical analyses.

Data were statistically analyzed using GraphPad Prism version 7.0 (GraphPad Software Inc., San Diego, CA, USA). The differences were analyzed using two-tailed unpaired Student’s *t* tests. Results are expressed as means ± standard deviation unless otherwise mentioned. Statistical significance was set at *P < *0.05.

### Data availability.

Genome sequences were submitted to GenBank and assigned the following accession numbers: CP038463 (WCX23, chromosome), CP038464 (WCX23, plasmid), CP038465 (23-C-23, chromosome), and CP038466 (23-C-23, plasmid). The nucleotide sequences of *envZ*, *ompR*, wild-type *mlaF*, *mlaF_D173A_*, and gene*3832* have been submitted to GenBank with the accession numbers MN862663, MN862664, MN862666, MN885538, and MT812950, respectively. The transcriptome sequencing (RNA-Seq) raw data were deposited in the NCBI Sequence Read Archive (SRA) with the accession numbers PRJNA705802 and PRJNA706457.
