# Supplementary material for: Various Novel Colistin Resistance Mechanisms Interact To Facilitate Adaptation of Aeromonas hydrophila to Complex Colistin Environments
Source: Antimicrob Agents Chemother. 2021 Jun 17;65(7):e00071-21. doi: 10.1128/AAC.00071-21 (PMC8373241; doi:10.1128/AAC.00071-21)
Supplement: Supplemental file 1 — Fig. S1, Fig S2, Table S1, Table S3, Table S4, Table S5. Download AAC.00071-21-s0001.pdf, PDF file, 0.5 MB [file aac.00071-21-s0001.pdf]

Supplementary data

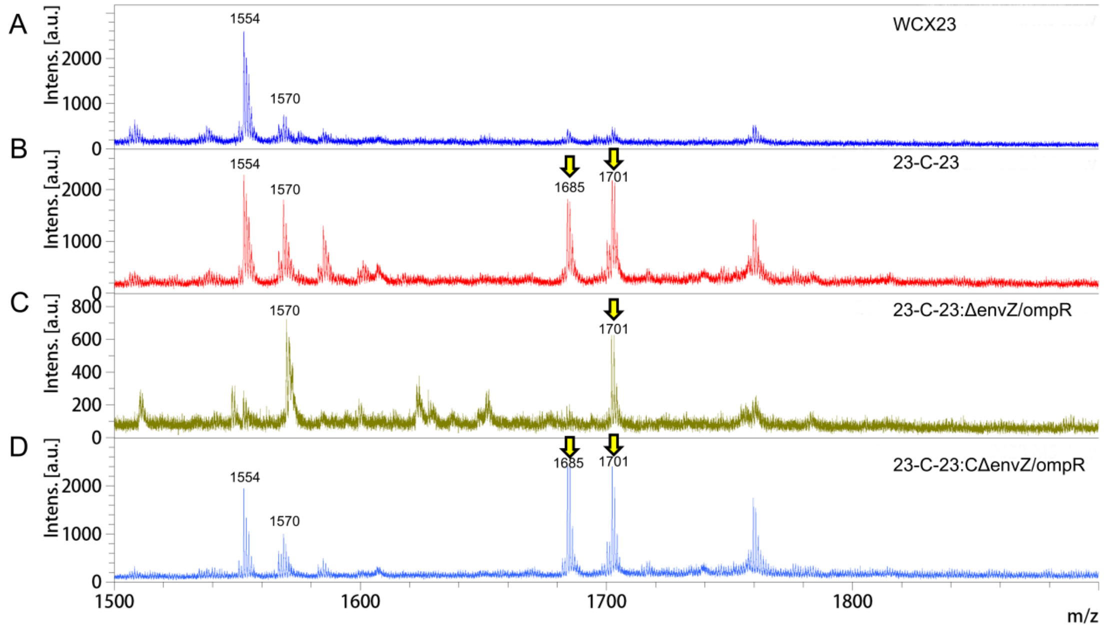

FIG S1 MALDI-TOF mass spectrometry of lipid A extracted from *A. hydrophila* strains. The x-axis represents the mass/charge ratio; the y-axis represents intensity (a.u.). Δm/z +131 indicates the addition of an L-Ara4N group. The L-Ara4N-modified lipid A species are denoted by yellow arrows. (A) The colistin-susceptible strain WCX23 showed a mass ion at m/z 1554 corresponding to a base lipid A structure that also existed with an additional hydroxylation (m/z 1570). (B) The colistin-resistant 23-C-23 showed additional ions at m/z 1658 and 1701, indicating an L-Ara4N addition to the base structures at m/z 1554 and 1570. (C) Absence of peaks at m/z 1554 and 1685 in 23-C-23:ΔenvZ/ompR. The intensity at m/z 1701 in 23-C-23:ΔenvZ/ompR (approximately 700) was significantly decreased compared to that in 23-C-23 (approximately 2200). (D) 23-C-23:CΔenvZ/ompR produced peaks at m/z 1554, 1570, 1658, and 1701, which were similar to those for colistin-resistant 23-C-23.

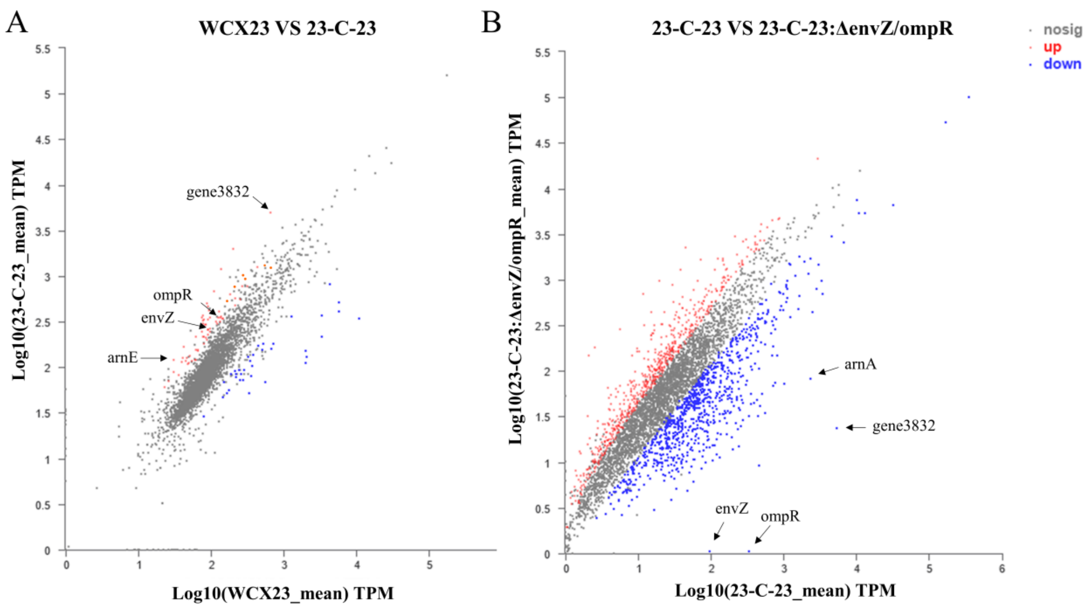

FIG S2 Scatter plot of expression changes. The x-axis and y-axis values, which are logarithmically treated, represent the gene expression levels in the two samples. Each dot represents a gene. Significantly upregulated genes are represented by red dots, significantly downregulated genes by blue dots, and non-significantly different genes by gray dots. Values approaching zero mean gene expression levels; the distance from the diagonal line means the degree of difference in gene expression between the two samples. (A) Scatter plot of expression changes between WCX23 (as the control group) and 23-C-23 (as the treatment group). Gene*3832* showed the third highest increase in expression in 23-C-23 compared to in WCX23. (B) Scatter plot of expression changes between 23-C-23 (as the control group) and 23-C-23:ΔenvZ/ompR (as the treatment group). Gene*3832* was the most downregulated gene in 23-C-23:ΔenvZ/ompR compared to in 23-C-23.

Table S1 Antimicrobial susceptibility of strains

	WCX23	23-C-23	23-C-23:ΔEnvZ/OmpR	23-C-23:CΔEnvZ/OmpR
Amoxicillin *	>4096; R	4096; R	2048; R	2048; R
Lincomycin *	512; R	512; R	512; R	256; R
Streptomycin *	4096; R	4096; R	>4096; R	4096; R
Spectinomycin *	>4096; R	4096; R	>4096; R	4096; R
Kanamycin *	1024; R	2048; R	1024; R	1024; R
Gentamicin *	16; R	16; R	16; R	16; R
Azithromycin *	32; R	16; R	16; R	16; R
Cefradine *	512; R	512; R	256; R	256; R
Ofloxacin *	0.25; S	0.5; S	0.25; S	0.25; S
Ciprofloxacin *	0.25; S	0.25; S	0.25; S	0.25; S
Aztreonam*	1; S	1; S	1; S	1; S
Sulfamethoxazole*	1024; R	1024; R	1024; R	2048; R
Ceftazidime*	4; S	4; S	2; S	2; S
Levofloxacin*	4; I	4; I	4; I	4; I

S, susceptibility; I, intermediate; R, resistant.

* All units are in mg/L

Table S3 Primer sequences for qRT-PCR

Gene	Primer F (5'-3')	Primer R (5'-3')
16s RNA	GAGATACGGGAGTGCCTTCG	GTGCTGGCAACAAAGGACAG
envZ	AAGGTCAGGGTCGAGATCCA	CATCCTCGATCTCGGTCAGC
ompR	TGACCCGTGAGAACTTCAGC	GTAATCGTCGGCACCCATCT
arnB	CGGATACCCTGATGACCAGC	CGTAGTGGACCGGGATGATG
arnC	AATACGGGCTGATGCACCTC	CGCATCAGGATCAGCAGGAT
arnA	GCTGGTGGAGAGCAAATCCT	GTTGCCGATGGTCTCTTCCA
arnD	ACTGAGGTAGGTCTGCGGAT	CCCCTTGACCTGATGCTTGT
arnT	ATCTGTTCGACAGCAAGGGG	TCGCGATCGGTCTTCATCAG
arnE	GCTCTGGATCTACCTGCTGC	TATGATGGCTCCCACTCCCA
arnF	ATGAGCAATGACCATCCGCA	GCCGAGCACGTAGAGATACC
gene*3832*	CGGTTTCGCTTCAACTTCCG	TCGCGGTTGTTGCTATCCTT
phoP	GTCAAGCCGTTCCAGAAGGA	GCGCATCAGGTATTCGAGGA
phoQ	AGGGACACCATCTGTCAGGA	CTCTCGCCGTTCTTGATGGT
pmrC	TGGTCAAGCCCAAGGATCAC	GCAGGAAATGGCGATGTGAC
mlaA	CTCGATCCTTACGCCCTGAC	CTTGCTTCTCCTGCTGCTCT
mlaB	CTGGTCAAATGGGCCAAAGC	AGCGGGGTTGACTGAAACAA
mlaC	TCAAGAAGATCGCCCTGCTG	TTGCTCTTCACCTGAGCCTG
mlaD	GACATCACTCTGGACGCCAA	TGGAGAGGGTACTGGTCTCG

mlaE	AGCAGTTGTATGTGGTCGGG	AGCAGAGAGAGGGAGACCAG
mlaF	CCGACCTGACCTTCAGTCAC	TGGGGATATCTTCCCCGTCA

Table S4 Primer sequences for allelic replacement of mlaF mutant

	Primer sequences (5'-3')	Description (5'-3')
mlaF-5F	GATCAGGCTGAACATGAAGGCCAG	For amplification of upstream homologous sequences
mlaF-5R	GTGAAGCTCATCAAGGATATGAACGATGC	
mlaF-3F	GAACGGCTCGTCATACATGATGAGG	For amplification of downstream homologous sequences
mlaF-3R	CTGCGACGAAAATCGAACAGTTCAG	
mlaF-sacB-F	GCATCGTTCATATCCTTGATGAGCTTCACCTAGGAGATCCTGGTATGACTAGTGC	For amplification of sacB-Apr sequences
mlaF-sacB-R	CCTCATCATGTATGACGAGCCGTTCCACATATACCTGCCGTTCACTATTATTTAGTG	

Table S5 Primer sequences for gene knockout and complementation

	Primer sequences（5'→3'）	Description
**Gene deletion**		
envZ/ompR-5F	GTCTCTTGGTCGGGTCCTGGTC	For amplification of upstream homologous sequences of envZ/ompR
envZ/ompR-5R	TGCTTGCCTCTCCTCTCGATATGC	
envZ/ompR-3F	GAGATCCGGCTGCCCAAGAAC	For amplification of downstream homologous sequences of envZ/ompR
envZ/ompR-3R	CTAGCGCCTTCAAGATCTCCCTCAC	
envZ/ompR-Cm-F	GCATATCGAGAGGAGAGGCAAGCACATAT GAATATCCTCCTTAGTTCCTATTC	For amplification of the chloramphenicol resistance gene
envZ/ompR-Cm-R	GTTCTTGGGCAGCCGGATCTCGAGCTGCTTC GAAGTTCCTA	
gene*3832*-5F	CAGATACTTGTAGCGTGAGTGGAAG	For amplification of upstream homologous sequences of gene3832
gene*3832*-5R	AAGTACAGCTTCTAATCATCCTTCCTG	
gene*3832*-3F	CAGAACCAGAACTGAAAGTGCAAC	For amplification of downstream homologous sequences of gene3832
gene*3832*-3R	GTCATCACCCTATGAAACCCTTGTC	
gene*3832*-Cm-F	CAGGAAGGATGATTAGAAGCTGTACTTTTA CGCCCCGCCCTGCCAC	For amplification of the chloramphenicol resistance gene
gene*3832*-Cm-R	GTTGCACTTTCAGTTCTGGTTCTGAGGAGCT AAGGAAGCTAAAATGGAG	
phoP/phoQ-5F	CAGTTCATCGGCATCACGGGTCC	For amplification of upstream homologous sequences of phoP/phoQ
phoP/phoQ-5R	GATCCAGGCTGTCAATTAGAGGGCTTATGA C	

phoP/phoQ-3F	TTCACCCTCGACCTGCCCTGAG	For amplification of downstream homologous sequences of phoP/phoQ
phoP/phoQ-3R	CAATAGCGAAGAAGAGGATGCGGTGC	
phoP/phoQ-Cm-F	GTCATAAGCCCTCTAATTGACAGCCTGGATCCATATGAATATCCTCCTTAGTTCCTATTC	For amplification of the chloramphenicol resistance gene
phoP/phoQ-Cm-R	GCTCAGGGCAGGTCGAGGGTGAAGAGCTGCTTCGAAGTTCCTA	
**Gene complementation**		
envZ/ompR-C-5F	GTCTCTTGGTCGGGTCCTGGTCAACG	For amplification of upstream homologous sequences containing envZ/ompR
envZ/ompR-C-5R	TCAATCAATCCACTCCAGCGGCTGGT	
envZ/ompR-C-3F	ACGAAGGGCGTATCTGTGGATACGCC	For amplification of downstream homologous sequences
envZ/ompR-C-3R	CTAGCGCCTTCAAGATCTCCCTCACCTACTTTG	
envZ/ompR-Apr-F	ACCAGCCGCTGGAGTGGATTGATTGATAATGACCCCGAAGCAGGGTTATG	For amplification of apramycin resistance gene
envZ/ompR-Apr-R	GGCGTATCCACAGATACGCCCTTCGTGGAATAGGAACTTATGAGCTCAGCCAA	
gene*3832*-C-5F	CAGATACTTGTAGCGTGAGTGGAAG	For amplification of upstream homologous sequences containing gene3832
gene*3832*-C-5R	TCATCCTTCCTGTAGTGTCTTCATTTTTAC	
gene*3832*-C-3F	CAGAACCAGAACTGAAAGTGCAAC	For amplification of downstream homologous sequences
gene*3832*-C-3R	GTCATCACCCTATGAAACCCTTGTC	
gene*3832*-Apr-F	GTAAAAATGAAGACACTACAGGAAGGATG	For amplification of apramycin

	AGGAATAGGAACTTATGAGCTCAGCCAA	resistance gene
gene*3832*-Apr-R	TAATGACCCCGAAGCAGGGTTATG	
